# Promising New Methods Based on the SOD Enzyme and SAUR36 Gene to Screen for Canola Materials with Heavy Metal Resistance

**DOI:** 10.3390/biology13060441

**Published:** 2024-06-17

**Authors:** Yue Dai, Hao Chen, Yufang Li, Rongkui Hui, Zhenqian Zhang

**Affiliations:** 1 College of Agriculture, Agricultural University of Hunan, 1 Agricultural Road, Changsha 410128, China; xgs623244682@stu.hunau.edu.cn (Y.D.); chenhao_sci@hunau.edu.cn (H.C.); 2 Hunan Cotton Science Institute, No. 3036 Shanjuan Road, Changde 415101, China; liyufang82@126.com; 3 Hunan Province Institute of Agricultural Science, South of Hongyuan East Road, Changsha 410125, China

**Keywords:** canola, germination, heavy metals, phytohormone

## Abstract

**Simple Summary:**

Canola is the largest self-produced vegetable oil source in China; however, excessive levels of cadmium, lead, and arsenic seriously affect its yield. In this study, canola near-isogenic lines with different oil contents (F338 (40.62%) and F335 (46.68%) as the control) were used as the experimental materials, and heavy metal stress experiments and omics analysis were carried out. The results show that superoxide dismutase and SAUR36 were closely related to heavy metal stress tolerance. Therefore, they may be used to screen for new canola materials with good heavy metal stress tolerance for canola breeding.

**Abstract:**

Canola is the largest self-produced vegetable oil source in China, although excessive levels of cadmium, lead, and arsenic seriously affect its yield. Therefore, developing methods to identify canola materials with good heavy metal tolerance is a hot topic for canola breeding. In this study, canola near-isogenic lines with different oil contents (F338 (40.62%) and F335 (46.68%) as the control) and heavy metal tolerances were used as raw materials. In an experiment with 100 times the safe standard values, the superoxide dismutase (SOD) and peroxidase (POD) activities of F335 were 32.02 mmol/mg and 71.84 mmol/mg, while the activities of F338 were 24.85 mmol/mg and 63.86 mmol/mg, exhibiting significant differences. The DEGs and DAPs in the MAPK signaling pathway of the plant hormone signal transduction pathway and other related pathways were analyzed and verified using RT-qPCR. SAUR36 and SAUR32 were identified as the key differential genes. The expression of the SAUR36 gene in canola materials planted in the experimental field was significantly higher than in the control, and FY958 exhibited the largest difference (27.82 times). In this study, SOD and SAUR36 were found to be closely related to heavy metal stress tolerance. Therefore, they may be used to screen for new canola materials with good heavy metal stress tolerance for canola breeding.

## 1. Introduction

In China, edible oil is in short supply [1,2], and canola is the largest source of edible vegetable oil. Thus, it is important to increase the planting area and yield of canola [3]. However, the point overshooting rate of Chinese arable land has reached 19.4% [4]. As the largest canola planting area in China, Hunan Province is deeply affected by excessive heavy metals [5], among which the levels of Cd, Pb, Hg, and As were measured as 7.0%, 1.5%, 1.6%, and 2.7%, respectively. Rice is difficult to sell due to excessive cadmium levels.

Heavy metals in soil lead to the production of secondary metabolites in plants and serious phytotoxicity [6], which seriously affects the growth, metabolism, physiology, and aging of plants [7]. Cd accumulation affects plants’ absorption of mineral elements [8], alters photosynthesis [9] and antioxidant enzyme activities [10], and even leads to death [11]. Pb affects the transport of plant materials [12] and negatively affects metabolic processes [13], causing delays in growth and germination [14]. As inhibits plant root growth and causes plant death [15]. Poor arable land quality significantly impacts the growth and development of canola [16], resulting in a poor canola yield. Therefore, screening materials with good heavy metal tolerance and studying their internal molecular mechanisms is a key topic of canola breeding research at present [17].

Heavy metals in soil may be easily absorbed by plant roots and transported to other organs and tissues, followed by oxidative stress and the production of related proteins and hormones, such as amino acids, antioxidants, and signaling molecules; compounds such as glutathione, plant chelate peptides, and metallothionein; and enzymes such as superoxide dismutase and peroxide [18]. Plants under metal stress are stimulated by antioxidant enzymes and related metabolic proteins, which play a vital role in signal transduction pathways [19]. Increases in ROS (reactive oxygen species) are considered to be the main phytotoxic effects of heavy metal stress [20,21]. With the development of molecular research technology, sequencing technologies, such as transcriptomics, metabolomics, proteomics, and genomics, and the combination of multiple analysis methods have been widely used [22,23]. For example, transcriptomics and genomics association analyses under different Cd tolerance levels revealed that the Nramp family genes were related to the transport of heavy metal ions in Arabidopsis thaliana, of which BnNramp2; 1 and BnNramp4; 2 were related to Cd transport [23]. Most of the previous studies were limited to a single molecular or physiological level [21,24]. However, the molecular mechanisms of canola’s heavy metal stress tolerance may be related to genes, proteins, and enzymes [25,26]; thus, there is an urgent need for comprehensive research on different aspects, such as the genome, the proteome, physiological characteristics, and field phenotypes [27,28,29].

In this study, canola near-isogenic lines (NILs) with significant phenotypic differences under heavy metal stress were used as the experimental materials, and transcriptomics and proteomics association analyses, verified using RT-qPCR, were used to examine the agronomic traits to identify the key genes or enzymes related to heavy metal stress tolerance and provide a reference for canola breeding.

## 2. Materials and Methods

### 2.1. Plant Materials

The canola NILs F335 and F338 were used as the raw materials and were provided by the College of Agriculture, Hunan Agricultural University. The materials had stable conventional propagation characteristics (Table S1).

### 2.2. Experimental Method

#### 2.2.1. Treatment Methods for Heavy Metal Stress

A mixture of three heavy metal standard solutions of Cd, Pb, and As was prepared for the heavy metal stress experiment, and the water culture germination method was adopted. The concentration of heavy metals was set as 10 times, 50 times, and 100 times the safe standard values of cadmium, lead, and arsenic in water (5 µg/kg, 20 µg/kg, and 10 µg/kg), referred to as 10×, 50×, and 100× in this study (Table 1). The canola in the indoor experiment was placed in a germination box for heavy metal stress growth. Fifty full and disease-free seeds were selected, soaked in 75% alcohol for 30 s for disinfection and washed with sterile deionized water, then soaked in the mixed solution of heavy metals (As, Cd, and Pb) for 12 h and placed in the germination box, and supplemented with heavy metal solution with the same concentration every day for 7 days. The experimental design is 16 h a day of illumination, 8 h of darkness, a temperature of 25 °C, and an illumination intensity of 2455 lux. The experimental method referred to Soares et al. [30] and Kania et al. [31].

For indoor experimental materials, we mainly recorded germination potential, germination rate, emergence rate, and biomass of seeds on the seventh day after germination.

The formula for germination potential is: germination potential (%) = number of seeds germinated on the 3rd day/total number of seeds × 100%.

The formula for germination rate is: germination rate (%) = number of seeds germinated on the 7th day/total number of seeds × 100%

The formula for emergence rate (%) = number of seeds emerged on the 7th day/total number of seeds × 100%.

#### 2.2.2. Physiological Indexes under Heavy Metal Stress

The seedlings on the 7th day of the 100× experiment were taken as samples to detect four enzymes, namely, superoxide dismutase (SOD), peroxidase (POD), catalase (CAT), and malondialdehyde (MDA). The SOD, POD, and CAT activities were determined according to Yang et al. [32] and Shi et al. [33], and the MDA content according to Draper et al. [34].

#### 2.2.3. Omics Analysis

The seedlings on the 7th day of the 100× experiment were selected as samples, washed with pure water, frozen in liquid nitrogen, stored at −80 °C, and then sent to BGI Gene and Hangzhou Jingjie Biotechnology Co., Ltd. (Hangzhou, China) for transcriptome and iTRAQ analysis.

A total of 276,873 chromatograms were obtained from proteome by mass spectrometry analysis, and 71,762 effective chromatograms were matched by Maxquant (v1.6.15.0), and the data were filtered by search database analysis. The accuracy FDR of spectrum, peptide, and protein identification was set at 1%. The identification protein needed to contain at least one unique peptide segment. We compared the relative quantitative values of protein for *t*-tests, and calculated the corresponding *p*-value as the significance index. The default *p*-value is ≤0.05. When the *p* value was less than 0.05, the differential expression level was significantly upregulated when it exceeded 1.3, and significantly downregulated when it was less than 1/1.3.

Transcriptome sequencing used fold change ≥ 2 and false discovery rate < 0.01 as the screening criteria for differential genes. FDR was obtained by correcting the difference significance *p*-value. In the correlation analysis between transcription group and protein group, when Log2 FC > 1 and the verification *p* value was less than 0.01, it was a significant difference expression of the transcript, and when Log2 FC < −1 and the verification *p* value was less than 0.01, it was a significant difference expression of the transcript. When the ratio was greater than 1.3 and the *p* value was less than 0.05, the upregulated protein was significantly differentially expressed. When the ratio was less than 1/1.3 and the *p* value was less than 0.05, the downregulated protein was significantly differentially expressed. The screening criteria for the results were the same as those used by Ye et al. [35].

#### 2.2.4. Quantitative Real-Time PCR (RT-qPCR) Detection

RNA was extracted and cDNA was reverse-transcribed using the TransZol Up Plus RNA kit (Beijing, China) and One-Step gDNA Removal (TRANS). The Hieff^®^ qPCR SYBR Green Master Mix (High Rox Plus) (Shanghai, China) was employed for the RT-qPCR. The parameter settings and gene expression calculations were the same as those used by Ye et al. [35].

#### 2.2.5. Field Experiment

Six canola varieties (FY730; FY737; FY823; FY958; ZY17; and SY664) were planted in the same field. They were transplanted into the field with Cd > 0.3 mg/kg (Q) and the control with Cd < 0.1 mg/k (Y) at the 5–6 leaf stage and then sampled three times: once every 14 d, i.e., at 14 days (A), 28 days (B), and 42 days (C), respectively, for RT-qPCR. The contents of heavy metals in the two fields are different (Table S3). Urea, KCL and potassium dihydrogen phosphate are used to supplement the nutrients, so as to ensure that the nutrients in the two places are consistent with the field management methods.

### 2.3. Data Analysis

Each result in this study was the average value of three replicates. IBM SPSS Statistics 25 statistical software (25.0) was used for the correlation analysis of the experimental data.

## 3. Results

### 3.1. Performance of Canola NILs at Different Heavy Metal Concentrations

**Figure 1:**
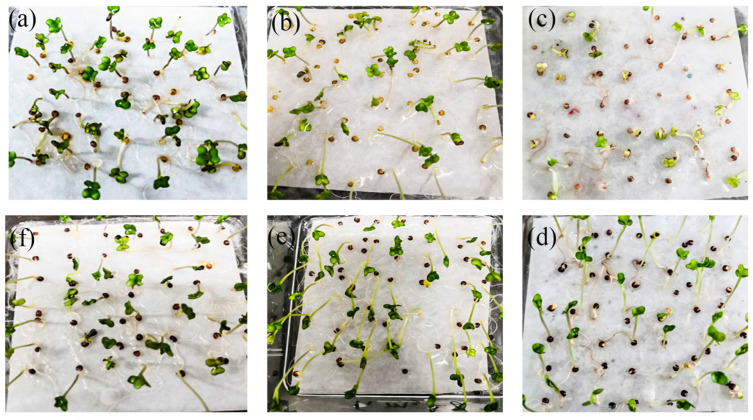
Seed germination at different heavy metal concentrations (50 seeds at each concentration). (**a**): F335 seed germination at 10× heavy metal concentration; (**b**): F335 seed germination at 50× heavy metal concentration; (**c**): F335 seed germination at 100× heavy metal concentration; (**d**): F338 seed germination at 10× heavy metal concentration; (**e**): F338 seed germination at 50× heavy metal concentration; (**f**): F338 seed germination at 100× heavy metal concentration.

The two canola NILs were treated with different heavy metal contents to compare their tolerance levels (Figure 1). The results show that at the 100× heavy metal concentration, the germination rate and biomass of F338 were significantly higher than those of F335. However, in the 50× heavy metal concentration experiment, the biomass of both materials exceeded 2.00 g, and the germination rate exceeded 98.00% (Table 2). The results show that low concentrations of heavy metals can promote seed germination, but higher concentrations may have a significant toxic effect on seed germination and root growth. F338 was minimally affected by heavy metal stress, which may be because it has a lower oil content and higher protein content, whereby the heavy metal ions form metal–protein complexes with the functional side-chain groups and are fixed, which reduces the toxicity of the metal ions to cells [36,37].

### 3.2. Physiological Performance of Canola NILs in Resisting Heavy Metal Stress

**Figure 2:**
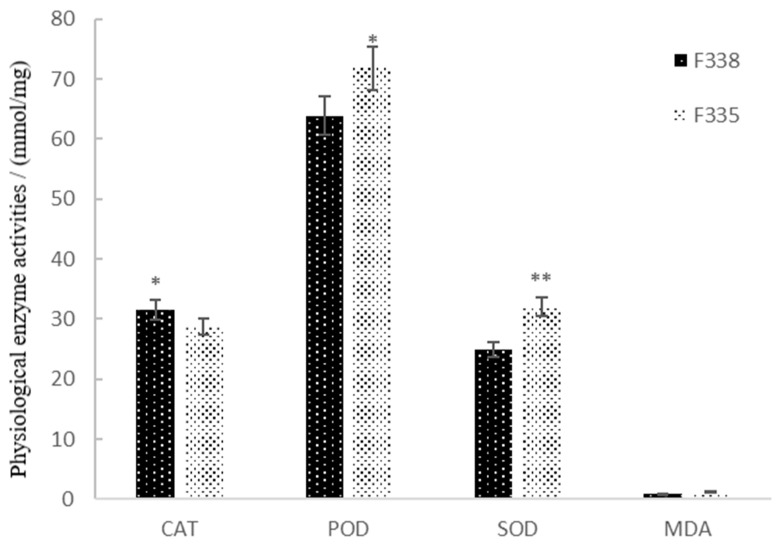
Physiological enzyme activities of the canola NILs under high-concentration heavy metal stress (100×). * Represents significant differences, ** represents extremely significant differences.

The physiological traits of the canola NILs under high-concentration heavy metal stress were compared (Figure 2). F335 and F338 exhibited significant differences in SOD and POD activities: 32.02 mmol/mg and 24.85 mmol/mg for SOD and 71.84 mmol/mg and 63.86 mmol/mg for POD, respectively. Under 100× heavy metal stress, the MDA contents also showed a significant difference of 1.34 times, indicating that heavy metal stress can cause lipid peroxidation. SOD and POD may be used as the main detoxification enzymes for canola seed germination subjected to heavy metal stress [38,39].

### 3.3. Omics Association Analyses of the Canola NILs in Response to Heavy Metals

The materials under 100× heavy metal stress were used for genomics and proteomics analyses.

#### 3.3.1. Transcriptome Analysis of Canola NILs under 100× Heavy Metal Stress

**Figure 3:**
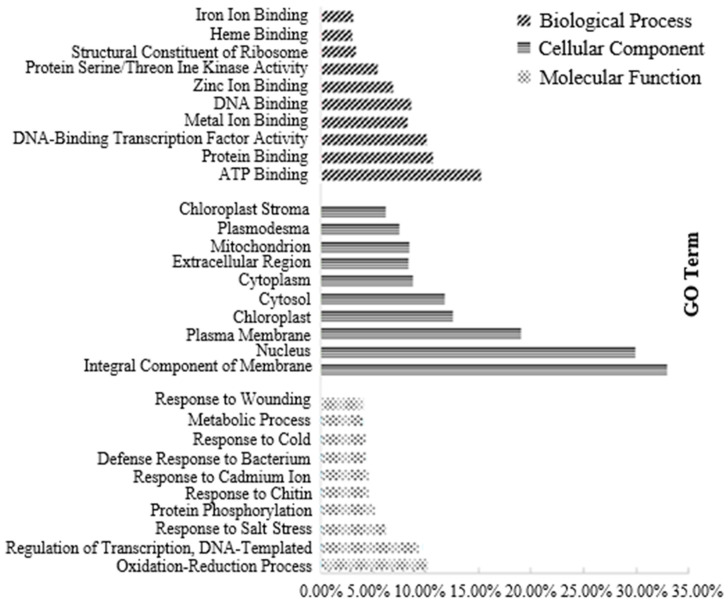
Differential genes of different materials in response to heavy metals in GO and COG analyses.

In total, 9665 DEGs were observed in the transcriptome analysis, of which 4820 genes were downregulated and 4845 were upregulated. The GO and COG analyses showed that the differential genes mainly participated in the redox process and the responses to cadmium ions, salt stress, and cold under high-concentration heavy metal stress, which indicated that the functional genes in canola that responded to heavy metal stress also participated in abiotic stress processes. The findings on protein, zinc ion, and iron ion binding from the molecular function (MF) and cellular component (CC) analysis indicate that heavy metal ions may enter through the inorganic salt absorption channels in canola and then selectively penetrate the cell membrane, which transfers and fixes them to vacuoles and the cytoplasm, thus reducing the toxicity of the heavy metals to the plant. Canola binds related proteins via activation, thus fixing the metal ions and reducing their toxicity (Figure 3). A total of 322 KEGG pathways were significantly enriched in KEGG, among which the most significant difference was in plant hormone signal transduction pathways [40]. The metabolism of starch and sucrose, protein processing in the endoplasmic reticulum, phenylpropionic acid biosynthesis, and plant–pathogen interactions were significantly different, which may explain the decrease in the germination rate and biomass of canola under heavy metal stress [41,42,43,44,45].

**Figure 4:**
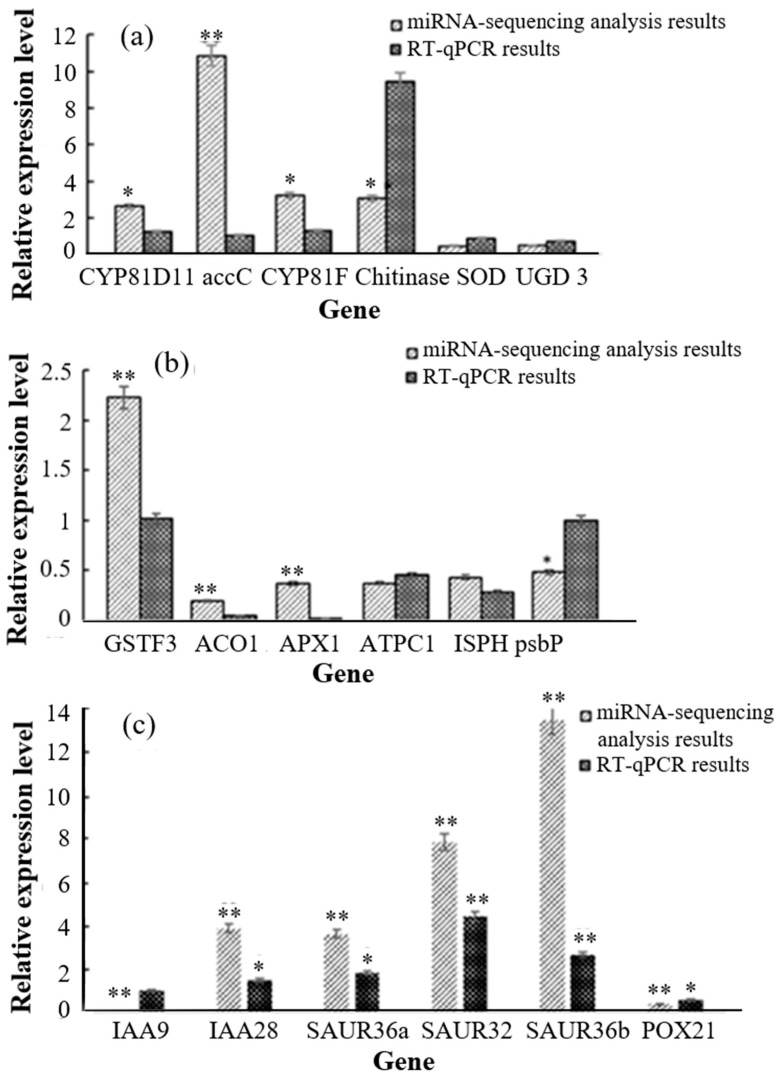
Expression of different differential genes. (**a**): RT-qPCR validation results for transcriptomics EDGs; (**b**): RT-qPCR validation results for proteomics DAPs (the corresponding genes); (**c**): transcriptomics and proteomics correlation analysis of differential gene expression. * Represents significant differences, ** represents extremely significant differences.

Six DEGs were selected and used to verify the results of the transcriptome analysis via RT-qPCR. The results were consistent with the transcriptomics data (Figure 4a), indicating that the transcriptome analysis results were reliable.

#### 3.3.2. iTRAQ Analysis of Canola NILs under 100× Heavy Metal Stress

**Figure 5:**
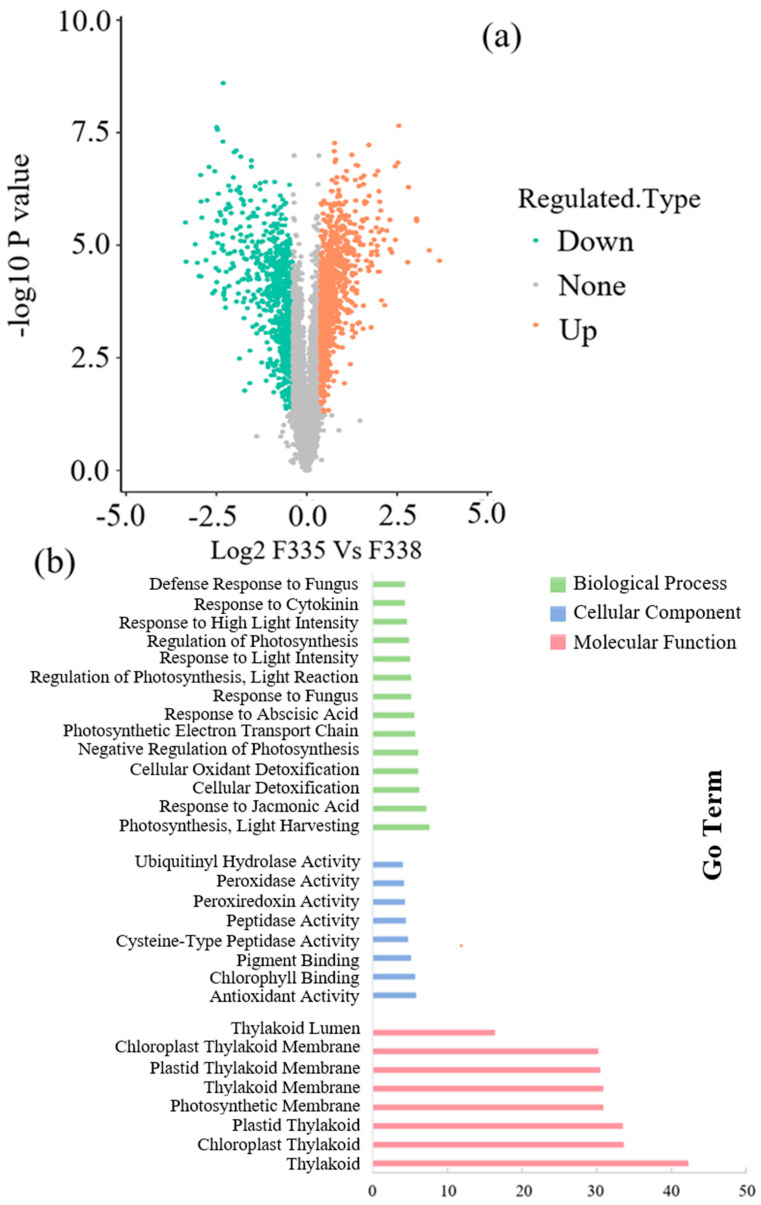
Differences in responses of different materials to heavy metals in proteomics results. (**a**): total DAPs in proteomics analysis; (**b**): DAPs in GO and COG analyses.

A total of 276,873 chromatograms were obtained from the iTRAQ analysis, 71,762 effective chromatograms were matched via MaxQuant analysis (v1.6.15.0), and 8925 proteins and 1787 differential proteins were identified (Figure 5a). The GO analysis showed that most DAPs in BP and MF were related to the cell interpretation of hormone antioxidant activity, and most were upregulated DAPs (Figure 5b). The downregulated DAPs mainly manifested as effects on seed and seedling development, further proving that heavy metal stress can cause membrane lipid peroxidation, affect life metabolism, and reduce biomass in plants. Twenty-six differential pathways were enriched in the KEGG pathway analysis. Compared with F335, F338’s downregulation of adenosine was mainly reflected in the degradation and synthesis of oil, indicating that heavy metal stress affects the substance synthesis of canola [41]. The KEGG pathways showed that the upregulated expression of DAPs was mainly focused on the pathways related to photosynthesis and plant hormones.

Six DAPs were selected, and their corresponding genes were used to verify the results of the proteomics analysis via RT-qPCR. The RT-qPCR results were consistent with the corresponding proteomics data (Figure 4b), indicating that the proteomics analysis results were reliable.

### 3.4. Validation of Transcription and Proteomics Analyses Using Real-Time Quantitative PCR (RT-qPCR)

In this study, 9665 DEGs and 1787 DAPs were identified by transcriptome and proteome, among which 183 DEGs and 11 DAPs were involved in plant hormone signal transduction and the MAPK signal pathway, and most of the related differential genes were members of the auxin response protein and peroxidase family. It has been found that KEGG pathways such as plant hormone signal transduction and the MAPK signal are highly correlated with plant resistance to heavy metals [40,46,47,48]. Comparing the differential genes in KEGG pathway, the expression of differential genes in the starch and sucrose metabolism pathway is greater, which shows that heavy metal stress can inhibit crop substance synthesis, destroy cell infiltration regulation, degrade protein hydrolysis activity, and finally, inhibit seed germination and seedling development [41,44,45]. In this study, under the stress of high concentration of heavy metals, seed germination was inhibited, and the time to enter the seedling stage after germination was prolonged, or even directly died, resulting in a decrease in biomass, which was consistent with the research results of Seneviratne and others [49]. In the early stage of seed germination, heavy metals inhibited the hydrolysis of carbohydrates and the transfer of hydrolyzed sugars, resulting in slow seedling growth [49].

Comprehensive analysis showed that genes with consistent expression trends in the transcriptome and proteome were mainly involved in pathways such as peroxisome metabolism, enzymatic activity, amino sugar and sugar metabolism, and startup and sugar metabolism. This indicates that crops can resist heavy metal stress and reduce its toxic effects by regulating the expression of key genes involved in metabolic processes, enzymatic activity, and signal transduction [50]. Heavy metal stress during plant growth affects related pathways such as biosynthesis, substance metabolism, and signal transduction (Table S2). In total, 23 DEGs and 27 DAPs were involved in phenylpropanoid biosynthesis, while 16 DEGs and 22 DAPs were involved in starch and sucrose metabolism. Plants respond to heavy metal stress via plant hormone signal transduction and the MAPK signaling pathway [50,51,52]. In the high-concentration heavy metal stress experiments, 56 DEGs in F338 and F335 were involved in plant hormone signal transduction, while 5 DEGs and 10 DAPs were involved in the MAPK signaling pathway (Table S2). The DEGs and DAPs in the related pathways were mainly members of the auxin-responsive protein and peroxidase families. Studies have shown that auxin-responsive protein and peroxidase are associated with plant resistance to heavy metal toxicity [53,54]. In the transcriptome analysis, 45 genes in the peroxidase (POX) family and 24 genes in the auxin-responsive protein ARF family were differentially expressed. Most of the genes in the auxin-responsive protein family were related to the expression of indole acetic acid (IAA) and SAUR. The SAUR-related genes and most of the 12 IAA-related genes were downregulated compared with the control group.

Five peroxidase genes were co-expressed in the transcriptome and proteome with significant differences. Two SAUR-related genes, two IAA-related genes, and one POX gene were examined using RT-qPCR. The results showed that the expression trends of the two SAUR-related genes BnaC04g00740D (SAUR32) and BnaC08g30850D (SAUR36) were the same in the transcriptome and proteome, and there were significant differences between F335 and F338, which indicated that they may be the key genes for plant resistance to heavy metal stress (Figure 4c). The small auxin-upregulated gene (SAUR) family is one of the main early auxin-responsive gene families found in higher plants and plays a central role in auxin-induced acidic growth. It can also be independently regulated by various other hormonal pathways and tissue-specific transcriptional factors [55,56]. SAUR36 is associated with senescence in plants [57,58], and its overexpression may cause slower hypocotyl growth and the disappearance of apical hooklet formation [59].

### 3.5. The Functional Verification of SAUR Genes

**Figure 6:**
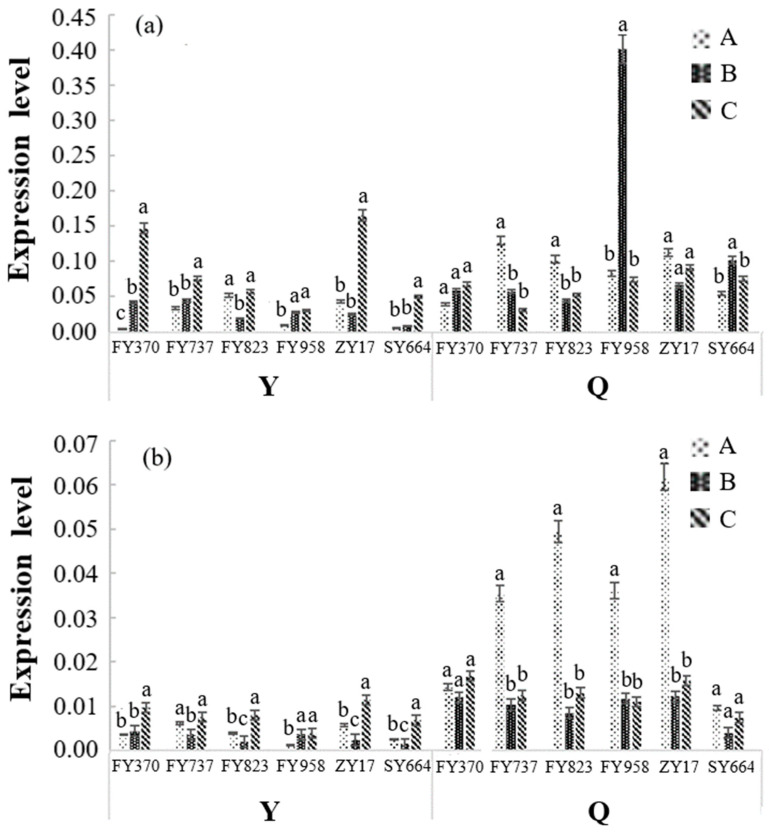
Validation of differential genes in Fields Y and Q (after the 5–6 leaf stage, samples were taken every 14 days, i.e., at 14 days (A), 28 days (B) and 42 days (C). (**a**): the expression level of SAUR32; (**b**): the expression level of SAUR36. a, b and c represent differences between different treatments.

The RT-qPCR analysis showed that in the first three periods, the expression level of SAUR36 in the sample from Field Q in Period A and the other periods was 27.82 times higher and more than 2.5 times higher than that in Field Y, respectively, especially in the canola of FY958 (Figure 6). A difference in SAUR36 expression of 1.07–13.20 times was observed in the other materials. This indicates that canola can tolerate heavy metal stress and maintain life activities by enhancing the expression of SAUR36 [55].

## 4. Discussion

### 4.1. Effects of Heavy Metal Stress on Physiological Performance of Canola

Upon entering plants, heavy metals promote the production of reactive oxygen species (ROS). The increase in the level of ROS leads to membrane lipid peroxidation and a large amount of O^2−^ accumulation in the cells, thus destroying the redox steady state of the cells [60,61] and plant metabolism and physiological responses [62,63]. This study found significant differences in SOD activity, POD activity, and MDA content between F335 and F338 under high-concentration heavy metal stress. The difference in SOD was the largest; therefore, SOD may be the key enzyme related to heavy metal stress. Plants can convert a large amount of O^2−^ produced via peroxidation into H_2_O_2_, which is then decomposed into H_2_O and O_2_, thus reducing the toxicity of heavy metals in the plants by enhancing SOD activity [38]. Studies have shown that SOD plays an important role in plant growth and development under stress [39,64,65,66,67]. In this study, SOD was the main heavy metal stress-resistant enzyme at the early growth stage of canola [68], and it mainly cooperated with POD in eliminating excessive ROS caused by heavy metal stress. Gokul et al. [68] found that SOD activity affected the heavy metal stress tolerance of Brassica napus. Yu et al. [69] revealed the function of SOD in the interaction between plants and abiotic stress, and SOD played an important role in plants’ tolerance to Cd stress. At present, SOD has been widely studied in the neighborhood where plants tolerate abiotic stress. For example, a whole-genome study identified that the SOD gene in canola was significantly expressed under abiotic stress [70]. Various stress-tolerant crops have been developed by modifying the SOD gene using transgenic methods [71]. SOD may be helpful for screening crops with heavy metal stress tolerance.

### 4.2. The Key Genes for Heavy Metal Stress Tolerance in Canola

A total of 183 DEGs and 11 DAPs involved in plant hormone signal transduction were found in this study. The MAPK signaling pathway was mostly related to the auxin-responsive protein and peroxidase family. Studies have shown that IAA can reduce the toxicity of heavy metals by reducing their absorption and increasing plant antioxidant capacity [54,72,73,74]. Overexpression of SAUR regulates cell wall acidification to induce plant growth [75,76]. Some histidine-rich regions at the N- and C-terminals of SAURs can also bind to metals [77] and may thus enhance plant environmental adaptability [45]. The expression of the SAUR gene is associated with tolerance to cold and salt stress [78]. SAURs are involved in the regulation of adaptive growth under abiotic stress and play an important role in plant adaptation to drought stress [79]. Current research shows the SAUR gene’s importance in regulating dynamic adaptive growth [55]. Qiu et al. [80] studied the function of the SAUR gene in Arabidopsis thaliana with CRISPR/Cas9 SAUR gene-editing technology, and the results showed that SAUR is an abscisic acid (ABA)-induced gene that regulates cell amplification, ion homeostasis, and plant salt tolerance. Many hormones and stress response elements exist in the promoter region of SAURs. The expression of SAURs may be induced by abiotic stress and exogenous hormones, which participate in the complex physiological processes in plants resisting abiotic stress [81]. SAUR36 has been found to play a vital role in plant senescence [58,82], regulating seed germination [83], promoting plant root growth [84], and enhancing plant resistance, such as salinity tolerance [84,85], waterlogging tolerance [86], etc. In this study, the expression of SAUR36 in the field was more significant than that of SAUR32, and the gene expression increased with the higher toxicity of heavy metals to canola, indicating that SAUR36 may play an important role in protecting plants from heavy metal stress. There is little research on the SAUR gene’s function in tolerating abiotic stress, and the research on SAUR36 mostly focuses on plant antiaging [87]. The results of this study show that SAUR36 may be a key gene for heavy metal stress tolerance in canola. Comparing the expression of SAUR36 in different materials may be a promising method to identify new materials with heavy metal stress tolerance for canola breeding.

## 5. Conclusions

In this study, the difference in the germination rate and biomass of the near-isogenic canola lines F335 and F338 under 100× heavy metal stress reached 1.69 times and 3.34 times. SOD activity was significantly different under high-concentration heavy metal stress, reaching 1.29 times. SOD might be the key enzyme for the early growth of canola while tolerating heavy metal toxicity. A total of 9665 EDGs and 1787 DAPs were obtained with transcriptome and proteome association analyses, respectively. The expressed DEGs and DAPs were mainly involved in the pathways related to photosynthesis, plant hormones, and plant hormone signal transduction, and most of them were members of the auxin-reactive protein and peroxidase family, especially the SAUR32 and SAUR36 genes. The expression level of SAUR36 in different canola materials was significantly different between the experimental field and the control, and the highest expression level difference was 27.82 times. The expression level of SAUR36 and activity of SOD may be useful for screening canola materials with heavy metal stress tolerance.

## Figures and Tables

**Table 1:** Concentration of heavy metal mixed solution.

Heavy Metal	Safety Standard (µg/kg)	10× (µg/kg)	50× (µg/kg)	100× (µg/kg)
Cd	5.00	50.00	250.00	500.00
Pb	20.00	200.00	1000.00	2000.00
As	10.00	100.00	500.00	1000.00

Note: The safety standard in the table is the standard value of heavy metal concentration in water.

**Table 2:** Germination of the near-isogenic materials under different heavy metal stress levels.

Materials	10×				50×				100×			
	A (%)	B (%)	C (%)	D (g)	A (%)	B (%)	C (%)	D (g)	A (%)	B (%)	C (%)	D (g)
F335	92.00	100.00	22.00	1.76	98.00	100.00	10.00	2.00	52.00	52.00	0.00	0.59
F338	90.00	100.00	20.00	2.38	96.00	98.00	0.00	2.43	92.00	88.00	0.00	1.97

Note: A: Germination potential of seeds on the third day after germination; B: Germination rate of seeds on the seventh day after germination; C: The emergence rate of seeds on the seventh day after germination; D: Biomass of the seventh day after seed germination.

## Data Availability

The study materials were provided by the College of Agriculture, at the Agricultural University of Hunan. The sequencing data have been deposited in the NCBI SRA database (accession number: PRJNA847036). The mass spectrometry proteomics data have been deposited in the Proteome X Change Consortium (dataset identifier: PXD035115).

## Supplementary Materials

The following supporting information can be downloaded at https://www.mdpi.com/article/10.3390/biology13060441/s1. Table S1: Quality of near-isogenic line materials and agronomic traits at different growth stages; Table S2: Analysis of omics differences under heavy metal stress; Table S3: Farmland soil data with different heavy metal contents.

## References

[1] Meilke K.D., Griffith G.R. (1981). An application of the Market Share Approach to the demand for soyabean and rapeseed oil. Eur. Rev. Agric. Econ..

[2] Shahid M., Cai G., Zu F., Zhao Q., Qasim M.U., Hong Y., Fan C., Zhou Y. (2019). Comparative Transcriptome Analysis of Developing Seeds and Silique Wall Reveals Dynamic Transcription Networks for Effective Oil Production in *Brassica napus* L.. Int. J. Mol. Sci..

[3] Yan L., Shah T., Cheng Y., LÜ Y., Zhang X.K., Zou X.L. (2019). Physiological and molecular responses to cold stress in rapeseed (*Brassica napus* L.). J. Integr. Agric..

[4] Deng J., Li W., Xu W., He Z., Tan X. (2021). Correlation and the concentrations of Pb, Cd, Hg and As in vegetables and soils of Chongqing, China. Environ. Geochem. Health.

[5] Guo Z.H., Song J., Xiao X.Y., Ming H., Miao X.F., Wang F.Y. (2010). Spatial distribution and environmental characterization of sediment-associated metals from middle-downstream of Xiangjiang River, southern China. J. Cent. South Univ. Technol..

[6] Asad S.A., Farooq M., Afzal A., West H. (2019). Integrated phytobial heavy metal remediation strategies for a sustainable clean environment-A review. Chemosphere.

[7] Munir O., Ersin Y., Salih G., Serdal Aksoy A. (2008). Plants as Biomonitors of Trace Elements Pollution in Soil. Trace Elements as Contaminants and Nutrients: Consequences in Ecosystems and Human Health.

[8] Liu J.G., Liang J.S., Li K.Q., Zhang Z.J., Yu B.Y., Lu X.L., Yang J.C., Zhu Q.S. (2003). Correlations between cadmium and mineral nutrients in absorption and accumulation in various genotypes of rice under cadmium stress. Chemosphere.

[9] Chandra R., Kang H. (2015). Mixed heavy metal stress on photosynthesis, transpiration rate, and chlorophyll content in poplar hybrids. For. Sci. Technol..

[10] Iannelli M.A., Pietrini F., Fiore L., Petrilli L., Massacci A. (2002). Antioxidant response to cadmium in Phragmites australis plants. Plant Physiol. Biochem..

[11] Yan H., Filardo F., Hu X., Zhao X., Fu D. (2016). Cadmium stress alters the redox reaction and hormone balance in oilseed rape (*Brassica napus* L.) leaves. Environ. Sci. Pollut. Res. Int..

[12] Dalyan E., Yüzbaşıoğlu E., Akpınar I. (2018). Effect of 24-Epibrassinolide on Antioxidative Defence System Against Lead-Induced Oxidative Stress in The Roots of *Brassica juncea* L. seedlings. Russ. J. Plant Physiol..

[13] Kohli S.K., Handa N., Sharma A., Gautam V., Arora S., Bhardwaj R., Alyemeni M.N., Wijaya L., Ahmad P. (2018). Combined effect of 24-epibrassinolide and salicylic acid mitigates lead (Pb) toxicity by modulating various metabolites in *Brassica juncea* L. seedlings. Protoplasma.

[14] Ye X.X., Wang G.Z., Zhang Y.X., Zhao H.J. (2018). Hydroxyapatite nanoparticles in root cells: Reducing the mobility and toxicity of Pb in rice. Environ. Sci. Nano.

[15] Meharg A.A., Hartley W.J. (2002). Arsenic uptake and metabolism in arsenic resistant and nonresistant plant species. New Phytol..

[16] Wei B., Yang L. (2010). A review of heavy metal contaminations in urban soils, urban road dusts and agricultural soils from China. Microchem. J..

[17] Wang Z., Yang C., Chen H., Wang P., Wang P., Song C., Zhang X., Wang D. (2018). Multi-gene co-expression can improve comprehensive resistance to multiple abiotic stresses in *Brassica napus* L.. Plant Sci..

[18] Hossain M.A., Piyatida P., Silva J.A.T., Fujita M. (2012). Molecular Mechanism of Heavy Metal Toxicity and Tolerance in Plants: Central Role of Glutathione in Detoxification of Reactive Oxygen Species and Methylglyoxal and in Heavy Metal Chelation. J. Bot..

[19] Shahid M., Dumat C., Khalid S., Schreck E., Xiong T., Niazi N.K. (2017). Foliar heavy metal uptake, toxicity and detoxification in plants: A comparison of foliar and root metal uptake. J. Hazard. Mater..

[20] Yu X.Z., Lin Y.J., Zhang Q. (2019). Metallothioneins enhance chromium detoxification through scavenging ROS and stimulating metal chelation in *Oryza sativa*. Chemosphere.

[21] Natasha S.M., Khalid S., Bibi I., Bundschuh J., Khan N.N., Dumat C. (2020). A critical review of mercury speciation, bioavailability, toxicity and detoxification in soil-plant environment: Ecotoxicology and health risk assessment. Sci. Total Environ..

[22] Wang S., Sun J., Li S., Lu K., Meng H., Xiao Z., Zhang Z., Li J., Luo F., Li N. (2019). Physiological, genomic and transcriptomic comparison of two *Brassica napus* cultivars with contrasting cadmium tolerance. Plant Soil..

[23] Guo J., Dai X., Xu W., Ma M. (2008). Overexpressing *GSH1* and *AsPCS1* simultaneously increases the tolerance and accumulation of cadmium and arsenic in *Arabidopsis thaliana*. Chemosphere.

[24] Xie T., Yang W., Chen X., Rong H., Wang Y., Jiang J. (2022). Genome-Wide Identification and Expressional Profiling of the Metal Tolerance Protein Gene Family in *Brassica napus*. Genes.

[25] Ding Y., Jian H., Wang T., Di F., Wang J., Li J., Liu L. (2018). Screening of candidate gene responses to cadmium stress by RNA sequencing in oilseed rape (*Brassica napus* L.). Environ. Sci. Pollut. Res. Int..

[26] Singh S., Parihar P., Singh R., Singh V.P., Prasad S.M. (2015). Heavy Metal Tolerance in Plants: Role of Transcriptomics, Proteomics, Metabolomics, and Ionomics. Front. Plant Sci..

[27] Adejumo S.A., Tiwari S., Thul S., Sarangi B.K. (2019). Evaluation of lead and chromium tolerance and accumulation level in Gomphrena celosoides: A novel metal accumulator from lead acid battery waste contaminated site in Nigeria. Int. J. Phytoremediation.

[28] Sruthi P., Puthur J.T. (2019). Characterization of physiochemical and anatomical features associated with enhanced phytostabilization of copper in *Bruguiera cylindrica* (L.) Blume. Int. J. Phytoremediation.

[29] Tang C., Zhang R., Hu X., Song J., Li B., Ou D., Hu X., Zhao Y. (2019). Exogenous spermidine elevating cadmium tolerance in Salix matsudana involves cadmium detoxification and antioxidant defense. Int. J. Phytoremediation.

[30] Soares T., Dias D., Oliveira A.M.S., Ribeiro D.M., Dias L. (2020). Exogenous brassinosteroids increase lead stress tolerance in seed germination and seedling growth of *Brassica juncea* L.. Ecotoxicol. Environ. Saf..

[31] Kania J., Krawczyk T., Gillner D.M. (2021). Oilseed rape (*Brassica napus*): The importance of aminopeptidases in germination under normal and heavy metals stress conditions. J. Sci. Food Agric..

[32] Yang P.M., Huang Q.C., Qin G.Y., Zhao S.P., Zhou J.G. (2014). Different drought-stress responses in photosynthesis and reactive oxygen metabolism between autotetraploid and diploid rice. Photosynthetica.

[33] Shi J., Fu X.Z., Peng T., Huang X.S., Fan Q.J., Liu J.H. (2010). Spermine pretreatment confers dehydration tolerance of citrus in vitro plants via modulation of antioxidative capacity and stomatal response. Tree Physiol..

[34] Draper H.H., Squires E.J., Mahmoodi H., Wu J., Agarwal S., Hadley M. (1993). A comparative evaluation of thiobarbituric acid methods for the determination of malondialdehyde in biological materials. Free Radic. Biol. Med..

[35] Ye S., Yan L., Ma X., Chen Y., Wu L., Ma T., Zhao L., Yi B., Ma C., Tu J. (2022). Combined BSA-Seq Based Mapping and RNA-Seq Profiling Reveal Candidate Genes Associated with Plant Architecture in *Brassica napus*. Int. J. Mol. Sci..

[36] Sharma S.K., Goloubinoff P., Christen P. (2008). Heavy metal ions are potent inhibitors of protein folding. Biochem. Biophys. Res. Commun..

[37] Tamas M.J., Sharma S.K., Ibstedt S., Jacobson T., Christen P. (2014). Heavy metals and metalloids as a cause for protein misfolding and aggregation. Biomolecules.

[38] Huang H.L., Rizwan M., Li M., Song F., Zhou S.J., He X., Ding R., Dai Z.H., Yuan Y., Cao M.H. (2019). Comparative efficacy of organic and inorganic silicon fertilizers on antioxidant response, Cd/Pb accumulation and health risk assessment in wheat (*Triticum aestivum* L.). Environ. Pollut..

[39] Nawaz M.A., Jiao Y.Y., Chen C., Shireen F., Zheng Z.H., Imtiaz M., Bie Z.L., Huang Y. (2018). Melatonin pretreatment improves vanadium stress tolerance of watermelon seedlings by reducing vanadium concentration in the leaves and regulating melatonin biosynthesis and antioxidant-related gene expression. J. Plant Physiol..

[40] Rahman S.U., Li Y.L., Hussain S., Hussain B., Khan W.D., Riaz L., Ashraf M.N., Khaliq M.A., Du Z.J., Cheng H.F. (2023). Role of phytohormones in heavy metal tolerance in plants: A review. Ecol. Indic..

[41] Adrees M., Ali S., Rizwan M., Ibrahim M., Abbas F., Farid M., Zia-Ur-Rehman M., Irshad M.K., Bharwana S.A. (2015). The effect of excess copper on growth and physiology of important food crops: A review. Environ. Sci. Pollut. Res. Int..

[42] Barceló J., Poschenrieder C. (1990). Plant water relations as affected by heavy metal stress: A review. J. Plant Nutr..

[43] Laetitia P.B., Nathalie L., Alain V., Cyrille F. (2002). Heavy metal toxicity cadmium permeates through calcium channels and disturbs the plant water status. Plant J..

[44] Karmous I., Bellani L.M., Chaoui A., Ferjani E., Muccifora S. (2015). Effects of copper on reserve mobilization in embryo of *Phaseolus vulgaris* L.. Environ. Sci. Pollut. Res. Int..

[45] Baszyński T. (2014). Interference of Cd^2+^ in functioning of the photosynthetic apparatus of higher plants. Acta Soc. Bot. Pol..

[46] Luo Z.B., Jiali He J.L., Polle A., Rennenberg H. (2016). Heavy metal accumulation and signal transduction in herbaceous and woody plants: Paving the way for enhancing phytoremediation efficiency. Biotechnol. Adv..

[47] Jalmi S.K., Bhagat P.K., Verma D., Noryang S., Tayyeba S., Singh K., Sharma D., Sinha A.K. (2018). Traversing the Links between Heavy Metal Stress and Plant Signaling. Front. Plant Sci..

[48] Li S.C., Han X.J., Lu Z.C., Qiu W.M., Yu M., Li H.Y., He Z.Q., Zhuo R.Y. (2022). MAPK Cascades and Transcriptional Factors: Regulation of Heavy Metal Tolerance in Plants. Int. J. Mol. Sci..

[49] Seneviratne M., Rajakaruna N., Rizwan M., Madawala H.M.S.P., Yong S.O., Vithanage M. (2017). Heavy metal-induced oxidative stress on seed germination and seedling development: A critical review. Environ. Geochem. Health.

[50] Chen K., Li G.J., Bressan R.A., Song C.P., Zhu J.K., Zhao Y. (2020). Abscisic acid dynamics, signaling, and functions in plants. J. Integr. Plant Biol..

[51] Kudla J., Batistic O., Hashimoto K. (2010). Calcium signals: The lead currency of plant information processing. Plant Cell.

[52] Thao N.P., Khan M.I., Thu N.B., Hoang X.L., Asgher M., Khan N.A., Tran L.S. (2015). Role of Ethylene and Its Cross Talk with Other Signaling Molecules in Plant Responses to Heavy Metal Stress. Plant Physiol..

[53] Kim Y.H., Lee H.S., Kwak S.S. (2010). Differential responses of sweetpotato peroxidases to heavy metals. Chemosphere.

[54] Nazli F., Wang X., Ahmad M., Hussain A., Bushra Dar A., Nasim M., Jamil M., Panpluem N., Mustafa A. (2021). Efficacy of Indole Acetic Acid and Exopolysaccharides-Producing Bacillus safensis Strain FN13 for Inducing Cd-Stress Tolerance and Plant Growth Promotion in *Brassica juncea* (L.). Appl. Sci..

[55] Stortenbeker N., Bemer M. (2019). The *SAUR* gene family: The plant′s toolbox for adaptation of growth and development. J. Exp. Bot..

[56] Hagen G., Guilfoyle T.J., Gray W.M. (2010). Auxin signal transduction. Plant Hormones.

[57] Bemer M., Van M.H., Muino J.M., Ferrandiz C., Kaufmann K., Angenent G.C. (2017). FRUITFULL controls *SAUR10* expression and regulates Arabidopsis growth and architecture. J. Exp. Bot..

[58] Hou K., Wu W., Gan S.S. (2013). *SAUR36*, a small auxin up RNA gene, is involved in the promotion of leaf senescence in Arabidopsis. Plant Physiol..

[59] Sun N., Wang J., Gao Z., Dong J., He H., Terzaghi W., Wei N., Deng X.W., Chen H. (2016). Arabidopsis SAURs are critical for differential light regulation of the development of various organs. Proc. Natl. Acad. Sci. USA.

[60] Verma S., Verma P.K., Chakrabarty D. (2019). Arsenic Bio-volatilization by Engineered Yeast Promotes Rice Growth and Reduces Arsenic Accumulation in Grains. Int. J. Environ. Res..

[61] Rui H., Chen C., Zhang X., Shen Z., Zhang F. (2016). Cd-induced oxidative stress and lignification in the roots of two *Vicia sativa* L. varieties with different Cd tolerances. J. Hazard. Mater..

[62] Farooq M.A., Gill R.A., Ali B., Wang J., Islam F., Ali S., Zhou W. (2016). Subcellular distribution, modulation of antioxidant and stress-related genes response to arsenic in *Brassica napus* L.. Ecotoxicology.

[63] Garg N., Singla P. (2011). Arsenic toxicity in crop plants: Physiological effects and tolerance mechanisms. Environ. Chem. Lett..

[64] Rizhsky L., Liang H., Mittler R. (2003). The water-water cycle is essential for chloroplast protection in the absence of stress. J. Biol. Chem..

[65] Myouga F., Hosoda C., Umezawa T., Iizumi H., Kuromori T., Motohashi R., Shono Y., Nagata N., Ikeuchi M., Shinozaki K. (2008). A heterocomplex of iron superoxide dismutases defends chloroplast nucleoids against oxidative stress and is essential for chloroplast development in Arabidopsis. Plant Cell.

[66] Basu U., Good A.G., Taylor G.J. (2001). Transgenic *Brassica napus* plants overexpressing aluminium-induced mitochondrial manganese superoxide dismutase cDNA are resistant to aluminium. Plant Cell Environ..

[67] Imtiaz M., Tu S., Xie Z., Han D., Ashraf M., Rizwan M.S. (2015). Growth, V uptake, and antioxidant enzymes responses of chickpea (*Cicer arietinum* L.) genotypes under vanadium stress. Plant Soil..

[68] Gokul A., Cyster L.F., Keyster M. (2018). Efficient superoxide scavenging and metal immobilization in roots determines the level of tolerance to Vanadium stress in two contrasting *Brassica napus* genotypes. S. Afr. J. Bot..

[69] Yu X.Z., Yang L., Feng Y.X. (2020). Comparative response of SOD in different plants against cadmium and drought stress at the molecular level. Appl. Environ. Biotechnol..

[70] Su W., Raza A., Gao A., Jiao Z.Q., Zhang Y., Hussain M.A., Mehmood S.S., Cheng Y., Lv Y., Zou X.L. (2021). Genome-Wide Analysis and Expression Profile of Superoxide Dismutase (SOD) Gene Family in Rapeseed (*Brassica napus* L.) under Different Hormones and Abiotic Stress Conditions. Antioxidants.

[71] Lee S.Y., Cheon K.S., Kim S.Y., Kim J.H., Kim W.H. (2020). Expression of sod2 enhances tolerance to drought stress in roses. Hortic. Environ. Biotechnol..

[72] Khare S., Singh N.B., Niharika Singh A., Amist N., Azim Z., Yadav R.K. (2022). Phytochemicals mitigation of *Brassica napus* by IAA grown under Cd and Pb toxicity and its impact on growth responses of Anagallis arvensis. J. Biotechnol..

[73] Ran J., Zheng W., Wang H., Wang H., Li Q. (2020). Indole-3-acetic acid promotes cadmium (Cd) accumulation in a Cd hyperaccumulator and a non-hyperaccumulator by different physiological responses. Ecotoxicol. Environ. Saf..

[74] Khan M.Y., Prakash V., Yadav V., Chauhan D.K., Prasad S.M., Ramawat N., Singh V.P., Tripathi D.K., Sharma S. (2019). Regulation of cadmium toxicity in roots of tomato by indole acetic acid with special emphasis on reactive oxygen species production and their scavenging. Plant Physiol. Biochem..

[75] Fendrych M., Leung J., Friml J. (2016). TIR1/AFB-Aux/IAA auxin perception mediates rapid cell wall acidification and growth of Arabidopsis hypocotyls. eLife.

[76] Spartz A.K., Lor V.S., Ren H., Olszewski N.E., Miller N.D., Wu G., Spalding E.P., Gray W.M. (2017). Constitutive Expression of Arabidopsis SMALL AUXIN UP RNA19 (*SAUR19*) in Tomato Confers Auxin-Independent Hypocotyl Elongation. Plant Physiol..

[77] Wu J., Liu S., He Y., Guan X., Zhu X., Cheng L., Wang J., Lu G. (2012). Genome-wide analysis of *SAUR* gene family in Solanaceae species. Gene.

[78] Kodaira K.S., Qin F., Tran L.S., Maruyama K., Kidokoro S., Fujita Y., Shinozaki K., Yamaguchi S.K. (2011). Arabidopsis Cys2/His2 zinc-finger proteins *AZF1* and *AZF2* negatively regulate abscisic acid-repressive and auxin-inducible genes under abiotic stress conditions. Plant Physiol..

[79] He Y., Liu Y., Li M., Lamin-Samu A.T., Yang D., Yu X., Izhar M., Jan I., Ali M., Lu G. (2021). The Arabidopsis SMALL AUXIN UP RNA32 Protein Regulates ABA-Mediated Responses to Drought Stress. Front. Plant Sci..

[80] Qiu T., Qi M.Y., Ding X.H., Zheng Y.Y., Zhou T.J., Chen Y., Han N., Zhu M.Y., Bian H.W., Wang J.H. (2020). The *SAUR41* subfamily of SMALL AUXIN UP RNA genes is abscisic acid inducible to modulate cell expansion and salt tolerance in *Arabidopsis thaliana* seedlings. Ann. Bot..

[81] Ma X.Q., Dai S.T., Qin N., Zhu C.C., Qin J.F., Li J.X. (2023). Genome-wide identification and expression analysis of the SAUR gene family in foxtail millet (*Setaria italica* L.). BMC Plant Biol..

[82] Huang C.K., Lo P.C., Huang L.F., Wu S.J., Yeh C.H., Lu C.A. (2015). A single-repeat MYB transcription repressor, MYBH, participates in regulation of leaf senescence in Arabidopsis. Plant Mol. Biol..

[83] Stamm P., Kumar P.P. (2013). Auxin and gibberellin responsive Arabidopsis SMALL AUXIN UP RNA36 regulates hypocotyl elongation in the light. Plant Cell Rep..

[84] Liu R., Wen S.S., Sun T.T., Wang R., Zuo W.T., Yang T., Wang C., Hu J.J., Lu M.Z., Wang L.Q. (2022). Pag*WOX11/12a* positively regulates the Pag*SAUR36* gene that enhances adventitious root development in poplar. J. Exp. Bot..

[85] Liu X., Liang W., Li Y.X., Li M.J., Ma B.Q., Liu C.H., Ma F.W., Li C.Y. (2019). Transcriptome analysis reveals the effects of alkali stress on root system architecture and endogenous hormones in apple rootstocks. J. Integr. Agric..

[86] Wang Y., Wang Y., Yang R., Wang F., Fu J., Yang W., Bai T., Wang S., Yin H. (2021). Effects of gibberellin priming on seedling emergence and transcripts involved in mesocotyl elongation in rice under deep direct-seeding conditions. J. Zhejiang Univ. Sci. B.

[87] Mahmood K., Kereamy A.E., Kim S.H., Nambara E., Rothstein S.J. (2016). *ANAC032* Positively Regulates Age-Dependent and Stress-Induced Senescence in *Arabidopsis thaliana*. Kashif Mahmood Plant Cell Physiol..

